# Predisposing Factors of Nosocomial Infections in Hospitalized Patients in the United Kingdom: Systematic Review

**DOI:** 10.2196/43743

**Published:** 2023-12-19

**Authors:** Sharon Shivuli Isigi, Ali Davod Parsa, Ibrahim Alasqah, Ilias Mahmud, Russell Kabir

**Affiliations:** 1 School of Allied Health Anglia Ruskin University Essex United Kingdom; 2 Department of Public Health College of Public Health and Health Informatics Qassim University Al Bukairiyah Saudi Arabia; 3 School of Health University of New England Armidale Australia; 4 BRAC James P Grant School of Public Health BRAC University Dhaka Bangladesh

**Keywords:** hospital-acquired infections, nosocomial infections, infection risk, systematic review, hospitalized patients, public health

## Abstract

**Background:**

Nosocomial infections are infections incubating or not present at the time of admission to a hospital and manifest 48 hours after hospital admission. The specific factors contributing to the risk of infection during hospitalization remain unclear, particularly for the hospitalized population of the United Kingdom.

**Objective:**

The aim of this systematic literature review was to explore the risk factors of nosocomial infections in hospitalized adult patients in the United Kingdom.

**Methods:**

A comprehensive keyword search was conducted through the PubMed, Medline, and EBSCO CINAHL Plus databases. The keywords included “risk factors” or “contributing factors” or “predisposing factors” or “cause” or “vulnerability factors” and “nosocomial infections” or “hospital-acquired infections” and “hospitalized patients” or “inpatients” or “patients” or “hospitalized.” Additional articles were obtained through reference harvesting of selected articles. The search was limited to the United Kingdom with papers written in English, without limiting for age and gender to minimize bias. The above process retrieved 377 articles, which were further screened using inclusion and exclusion criteria following the PRISMA (Preferred Reporting Items for Systematic Reviews and Meta-Analyses) guidelines. The retained 9 studies were subjected to critical appraisal using the Critical Appraisal Skills Programme (cohort and case-control studies) and Appraisal Tool for Cross-Sectional Studies (cross-sectional studies) checklists. Finally, 6 eligible publications were identified and used to collect the study findings. A thematic analysis technique was used to analyze data extracted on risk factors of nosocomial infections in hospitalized patients in the United Kingdom.

**Results:**

The risk factors for nosocomial infections that emerged from the reviewed studies included older age, intrahospital transfers, cross-infection, longer hospital stay, readmissions, prior colonization with opportunistic organisms, comorbidities, and prior intake of antibiotics and urinary catheters. Nosocomial infections were associated with more extended hospital stays, presenting with increased morbidity and mortality. Measures for controlling nosocomial infections included the use of single-patient rooms, well-equipped wards, prior screening of staff and patients, adequate sick leave for staff, improved swallowing techniques and nutritional intake for patients, improved oral hygiene, avoiding unnecessary indwelling plastics, use of suprapubic catheters, aseptic techniques during patient care, and prophylactic use.

**Conclusions:**

There is a need for further studies to aid in implementing nosocomial infection prevention and control.

## Introduction

Nosocomial infections are recognized public health issues worldwide with a prevalence of 3.0%-20.7% and an incidence rate of 5%-10% [1]. These infections are not present or incubating at the time of admission to a hospital but are acquired after hospitalization and manifest 48 hours after admission; 30% of nosocomial infections are estimated to be preventable [2]. Nosocomial infections are a potential risk to the patients, staff, and community, with many of the microorganisms isolated known to be drug-resistant, resulting in increased admissions associated with a longer hospital stay, mortality, and increased hospital expenses [3]. A recent study [4] found that nosocomial infections account for 80% of all hospital infections, which include catheter-associated urinary tract infections (UTIs), surgical site infections (SSIs), ventilator-associated pneumonia, hospital-acquired pneumonia, and *Clostridium difficile* infections. Major symptoms of nosocomial infections are productive cough, abdominal pain, shortness of breath, altered mental status, rebound tenderness, palpitations, suprapubic pain, dysuria, polyuria, and costovertebral angle tenderness [2].

The risk factors associated with nosocomial infections are dependent on infection control procedures at the facility, the prevalence of pathogens in the community, and patients’ immune status [1]. Research-based known risk factors include immunosuppression, older age, period of hospitalization, underlying comorbidities, frequency of hospital visits, mechanical ventilator support, invasive procedures, indwelling devices, longer stay in the intensive care unit (ICU), lack of infection control measures, and environmental hygiene [5]. The increased risk of nosocomial infections in older patients is due to frailty, suppressed immunity, and preexisting conditions [6].

Nosocomial outbreaks can be a cause of hospital closure, increased morbidity, and mortality. Therefore, to prevent and minimize nosocomial infections, several hospitals have implemented infection control procedures such as disinfection, steaming, proper waste management, isolation of affected staff until 48 hours after recovery, and aseptic hand washing before and after patient care, alongside prior identification of patients at risk of nosocomial infections and multidrug resistance [5].

Freeman and McGowan [6] found that risk factor identification allows for evaluating per-patient component risk, epidemiologic comparisons between hospital populations, and cost-effectiveness of infection prevention and control interventions. Calculation of risk per admission justifies findings on length of hospital stay as an increased risk for nosocomial infections [7]. This calculated per-day risk will account for the daily overall risk of infection per patient [6].

This systematic review was conducted to fill the gap in the absence of reviews on risk factors for nosocomial infections in hospitalized patients in the United Kingdom without limiting age and gender. The unique health care system in the United Kingdom funded by the National Health Service (NHS) is always at the frontline of adopting innovations and technologies in health care, which provides a guide to the international community on emerging innovations around infection control and prevention. Therefore, understanding predisposing factors of nosocomial infections in the United Kingdom can help inform local policies and practices, serving as a reference point for other countries considering revising their health care guidelines and policies.

The aims and objectives of this systematic review were focused on exploring the risk factors of nosocomial infections in hospitalized patients in the United Kingdom, the population at risk of nosocomial infections, impacts associated with nosocomial infections, and organisms associated with the spread of nosocomial infections.

## Methods

### Search Strategy

The search strategy was formulated based on the Population, Exposure, and Outcome framework, which is suitable for investigating the likelihood of developing a condition in the presence of a factor and providing practical strategies for controlling the outcome [8].

The PubMed and Google Scholar databases were searched for any existing or ongoing systematic reviews published between 2017 and 2021 regarding risk factors of nosocomial infections in hospitalized patients. Although systematic reviews were identified, none of these focused on the risk factors of nosocomial infections in hospitalized patients.

The review was focused on a comprehensive search using the PRISMA (Preferred Reporting Items for Systematic Reviews and Meta-Analyses) guidelines for systematic reviews of 2020 [9]. Headings and keywords were used through a database search, including the PubMed, EBSCO CINAHL Plus, and EBSCO Medline databases. Although searching multiple databases identified duplicate studies, this approach reduced the chances of missing out on important publications.

Boolean operators “OR” and “AND” were utilized to translate the research question into research strings to retrieve relevant and focused results. Medical Subject Headings in databases and keywords from other research papers were used to identify synonyms of the keywords for obtaining more focused results. The keywords included “risk factors” OR “contributing factors” OR “predisposing factors” OR “cause” OR “vulnerability factors” AND “nosocomial infections” OR “hospital-acquired infections” AND “hospitalized patients” OR “inpatients” OR “patients” OR “hospitalized.” Additional articles were obtained through reference harvesting of selected articles. The search was limited to the United Kingdom with papers in English, without limiting for age and gender to minimize bias.

### Study Selection

The inclusion and exclusion criteria for the systematic review are shown in Table 1.

**Figure 1:**
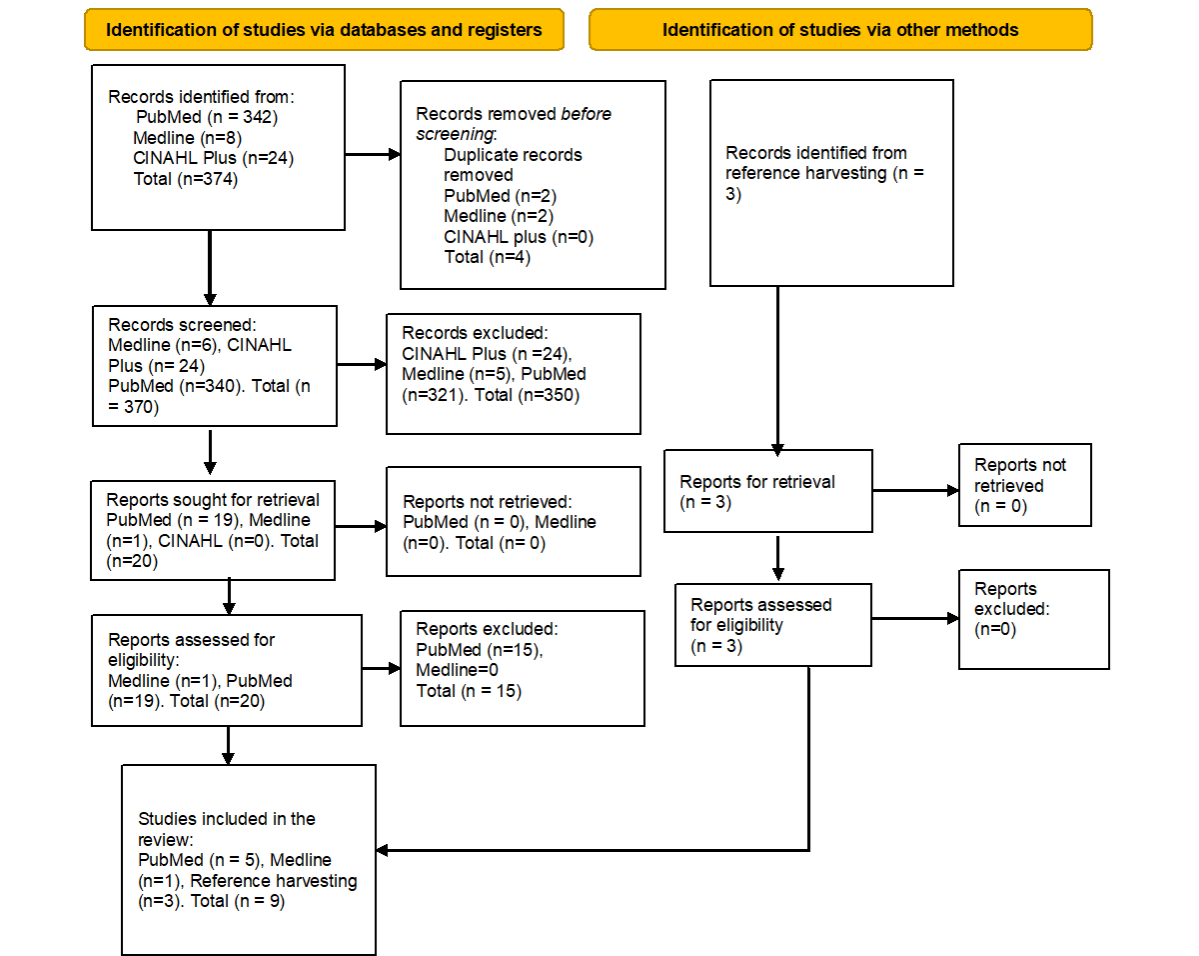
PRISMA (Preferred Reporting Items for Systematic Reviews and Meta-Analyses) 2020 flow diagram for study selection.

The database search resulted in 374 articles plus 3 articles retrieved from reference harvesting (Figure 1).

**Table 1:** Inclusion and exclusion criteria.

Category	Inclusion criteria	Exclusion criteria
Study type	Primary research studies, 2011-2021 time frame, peer-reviewed articles, English language, full-text articles, qualitative and quantitative studies	Systematic reviews, non-English language, articles published before 2011, not peer-reviewed articles, abstract only
Population	Hospitalized patients/patients/inpatients in the United Kingdom	Hospitalized patients outside the United Kingdom
Exposure	All hospitals in the United Kingdom	Hospitals outside the United Kingdom
Outcome	Nosocomial infections in the United Kingdom	Any other articles that did not collect information on nosocomial infections in the United Kingdom

RefWorks software was used to remove duplicate articles and titles were read manually to avoid duplication bias, resulting in 370 articles from all databases.

Title and abstract screening based on the inclusion and exclusion criteria resulted in 20 included articles and 350 excluded articles. The reasons for exclusion were that titles and abstracts were not giving relevant information on risk factors of nosocomial infections, guidelines, providing information on community-acquired infections in non-UK countries, or systematic reviews. Full-text article screening resulted in zero articles being excluded. In addition, 15 studies were excluded because of providing inadequate information on risk factors of nosocomial infections. After applying these criteria, nine articles, including the three obtained from reference harvesting, were subject to critical appraisal. A scoping review was carried out and no relevant papers were identified before 2011.

### Data Extraction

The data extracted were tabulated using Microsoft Word. The data drawn out included authors’ details, study design, the context of the study, sample size, aim of the study related to the risk of nosocomial infections, key findings, limitations, and conclusions of the identified studies.

### Data Analysis

Thematic analysis was conducted on the included studies following the Braun and Clarke [10] guidelines, who describe a thematic analysis as a useful and flexible method for qualitative research. Thematic analysis classifies themes and patterns that connect to data, enabling researchers to identify and detect variables influencing any issues in study participants [10]. The themes were identified by one author and then agreed upon via discussion with all authors.

### Critical Appraisal

Critical appraisal was applied to the selected 9 articles to check for the validity of the research if the results provided answers to the research question and the relevance of the information while minimizing bias, with the answers being either yes (+), no (–), or can’t tell (+/–). Included case-control (Table 2) and cohort (Table 3) studies were appraised using the Critical Appraisal Skills Programme (CASP) tool and cross-sectional studies (Table 4) were appraised using the Appraisal Tool for Cross-Sectional Studies (AXIS) checklist. The CASP tool is a checklist that uses focused questions to help researchers evaluate the validity and reliability of identified research articles. This provides appraisal skills and tools that facilitate checking research resources for trustworthiness, relevance, and results [11]. The AXIS tool was used to evaluate the reporting quality, study design, and risk of bias in cross-sectional studies [12]. This tool focuses on presented methods and results of studies, which helps researchers assess whether the findings are reliable and credible and whether they relate to the aims, methods, and analysis of what is reported in identified studies. Following critical appraisal, three articles were excluded due to lack of ethical approval, low internal validity, or reliability of results, resulting in a total of six included articles.

**Table 2:** Critical appraisal of the selected case-control studies using the Critical Appraisal Skills Programme tool [11].

Critical appraisal questions	Boncea et al [13]	Alrawi et al [14]	Ousey et al [15]
Did the study address a clearly focused issue?	+^a^	+	+
Did the authors use an appropriate method to answer their question?	+/–^b^	+	+/–
Were the cases recruited acceptably?	+	+	+/–
Were the controls selected acceptably?	+	+	+/–
Was the exposure accurately measured to minimize bias?	+/–	+	+
Aside from the experimental intervention were the groups treated equally?	+	+/–	+/–
Have the authors taken into account the potential confounding factors in the design and/or in their analysis?	+/–	–^c^	+/–
How large was the treatment effect?	+	–	+/–
How precise was the estimate of the treatment effect?	+	–	+
Do you believe the results?	+/–	–	+/–
Can the results be applied to the local population?	–	+/–	+/–
Do the results of this study fit with other available evidence?	+	+	+/–

^a^+:Yes.

^b^+/–:Unclear.

^c^–:No.

**Table 3:** Critical appraisal of the selected cohort studies using the Critical Appraisal Skills Programme tool [11].

Critical appraisal questions	Mo et al [16]	Behar et al [17]	Ewan et al [18]	Melzer and Welch [19]	Wloch et al [20]
Did the study address a clearly focused issue?	+^a^	*+*	*+*	*+*	*+*
Was the cohort recruited acceptably?	*+*	*+/*–^b^	*+/–*	*+*	*+*
Was the exposure accurately measured to minimize bias?	*+/*–	*+*	*+*	*+*	*+*
Was the outcome accurately measured to minimize bias?	*+/–*	*+*	*+/*–	*+*	*+/*–
Have the authors identified all important confounding factors?	*+*	*+*	*+*	*+*	*+*
Have they taken into account the confounding factors in the design and/or analysis?	*+*	*+*	*+*	*+*	*+/*–
Was the follow-up of subjects complete enough?	*+*	*+*	*+*	*+*	*+*
Was the follow-up of subjects long enough?	*+*	*+*	*+*	*+*	*+/*–
What are the results of the study?	*+*	*+*	*+*	*+*	*+*
How precise are the results?	*+*	*+*	*+*	*+*	*+*
Did you believe the results?	*+*	*+*	*+*	*+*	*+*
Can the results be applied to the local population?	*+*	*+*	*+/*–	–^c^	*+*
Do the results of this study fit with other available evidence*?*	*+*	*+*	*+/*–	*+*	*+*
What are the implications of the study for practice?	*+*	*+*	*+*	*+*	*+*

^a^+:Yes.

^b^+/–:Unclear.

^c^–:No.

**Table 4:** Critical appraisal of the selected cross-sectional study using the Appraisal Tool for Cross-Sectional Studies [12].

Critical appraisal questions	Ledwoch et al [21]
Was the aim/objective of the study clear?	+^a^
Was the study design appropriate for the stated aim?	+
Was the sample size justified?	+
Was the reference population clearly defined? (Is it clear who the research was about?)	+
Was the sample frame taken from an appropriate population to closely represent the reference population under investigation?	+
Was the selection process likely to select subjects/participants that were representative of the target/reference population under investigation?	+
Were measures undertaken to address and categorize nonresponders?	–^b^
Were the risk factor and outcome variables measured appropriate to the aim of the study?	+
Were the risk factor and outcome variables measured correctly using instruments/measurements that had been trialed, piloted, or published previously?	+
Were the methods (including statistical methods) sufficiently described to enable them to be repeated?	–
Were the basic data adequately described?	+
Does the response rate raise concerns about nonresponse bias?	+
If appropriate, was information about nonresponders described?	–
Were the results internally consistent?	+
Were the results presented for all the analyses described in the methods?	+
Were the authors’ discussion and conclusions justified by the results?	+
Were the limitations of the study discussed?	+
Was there funding or conflicts of interest that may affect the authors’ interpretation of results?	+
Was ethical approval or consent of participants attained?	–

^a^+:Yes.

^b^–:No.

## Results

### Characteristics of Included Studies

This systematic review identified 5 cohort studies [16-20], 3 case-control studies [13-15], and 1 cross-sectional study [21]. After critical appraisal, 5 cohort studies [16-20] and 1 case-control study [13] were included for analysis. The included studies covered countries within the United Kingdom, with the majority covering England. Most of the studies identified older adults and patients with preexisting conditions at increased risk of nosocomial infections. The organisms associated with increased nosocomial infections according to the majority of the included studies were *C. difficile*, *Escherichia coli*, *Pseudomonas aeruginosa*, *Enterococcus* spp, *Klebsiella pneumoniae*, *Staphylococcus aureus*, coliform species, anaerobic cocci, methicillin-resistant *S. aureus* (MRSA), coagulase-negative *Staphylococcus*, Enterobacteriaceae, and streptococci (Table 5).

**Table 5:** Data extracted from the included studies.

Reference	Study design	Context	Sample size	Study aim	Key findings	Limitations	Conclusion
Alrawi et al [14]	Case-control study	United Kingdom	Adult patients admitted to a burn center over 12 months	Determine the presence of different microbes in a UK burn center, and examine the relationships between bacterial colonization, burn size, length of hospital stay, and delayed referral	*Staphylococcus aureus* was common with 79% positive patients, and a direct link was found between an increased incidence of bacteria colonization, delay in referral >24 hours, length of hospital stay, and large burn size	None	A burn wound creates a good medium for bacteria colonization and proliferation; in understanding the sources of bacteria and patients’ susceptibility, wound care clinicians can form better management and treatment to reduce mortality and morbidity from burn wound sepsis
Behar et al [17]	Cohort study	England	727 patients admitted to an elderly medicine ward	Establish the risk factors for *Clostridium difficile* in hospitalized patients	9.8% of patients carried toxigenic *C. difficile* and ribotype 027 was not identified, while ribotype 106 was identified 3 times and in 7 others twice. Independent factors of colonization included previous *C. difficile* infection (OR^a^ 4.53, 95% CI 15.48) and malnutrition (OR 3.29, 95% CI 1.47-7.35)	Overestimation of carriers because of a higher rate in sampled patients than in unsampled patients. No certainty whether the infection was community-acquired or hospital-acquired. The study may be prone to type 2 errors. Failure to calculate sample size due to lack of information on the frequency of colonization before the start of the study.	The study identifies the common carriage of *C. difficile* in older patients. Patients with previous *C.* *difficile* infections are to be evaluated as a cost-effective intervention to reduce symptomatic *C. difficile* infection in hospitalized patients.
Boncea et al [13]	Case-control study	United Kingdom	3 hospitals with 24,240 older patients (>65 years)	Explore how intrahospital transfer influences the odds of developing an HAI^b^ in an urban hospital network	72.2% of the hospital transfer cases had at least one intrahospital transfer and each additional transfer increased the odds of acquiring a nosocomial infection by 9% in older patients (OR 1.09, 95% CI 1.05-1.13)	Lack of physiological data. Use of OPCS-4^c^, which lacks hierarchy in the invasiveness of procedures and failure to record minor medical devices. Unavailable information on prescribed antibiotics and proton pump inhibitors may result in confounding effects. Lack of staffing level; unclear if infection spread is due to staff or patient casual movement. Missing symptom information. Findings not generalizable to the younger population. Lack of time stamp inaccuracies and diagnostic coding errors.	Intrahospital transfers increased the odds of developing nosocomial infections. Strategies for minimizing transfer should be considered. Further studies needed to identify unnecessary transfers.
Ewan et al [18]	Cohort study	Northeast England	90 patients with lower limb fractures aged 65-101 years in a general hospital	Investigate the association between hospital-acquired pneumonia preceding heavy dental plaque and oral carriage of respiratory pathogens in older patients with limb fractures to determine the target for intervention studies	Hospital-acquired pneumonia was not associated with dentate, tooth number, or heavy dental plaques, but was associated with increased hospital stay and prior oral carriage of *Escherichia coli/S. aureus/Pseudomonas aeruginosa*/MRSA^d^ (OR 9.48, 95% CI 2.28-38.78; *P*=.002)	Results are not generalizable to medical patients. Biased sample toward “well patients,” leading to underestimation of exposure and outcome variables. Overestimated hospital-acquired pneumonia incidence. Overprediction of significance when the colonization index was larger than zero.	Lower limb fracture patients colonized with *E. coli*, *S. aureus*, MRSA, or *P. aeruginosa* after 5 days in hospital were at greater risk of hospital-acquired pneumonia (*P*=.002). Methods to implement and deliver good oral hygiene at the ward level in the health system need to be investigated to minimize the use of antibiotics and length of hospital stay.
Ledwoch et al [21]	Cross-sectional study	Wales, Scotland, England	3 hospitals and 1 dental practice	Investigate the presence of DSBs^e^ on 52 routinely cleaned keyboards from 4 hospitals across the United Kingdom	Hospital keyboards outside the patient area harbor DSBs, representing potential reservoirs for transferrable pathogens with 31% vancomycin-resistant *Enterococcus*, 17% MDR^f^ *Acinetobacter* spp, and 72% MRSA recovered from almost half of the 45 samples	Selective plates and quality control were not selective to a single species from the DSB	Hospital keyboards are a source of infection for HAIs with a need for further studies on DSBs to find products that can control DSBs while effectively preventing bacteria transfer
Melzer and Welch [19]	Cohort study	London	500 patients in Royal London Hospital	To determine host factors that can predict severe sepsis in a bacteremic cohort	Bacteremia largely occurred in patients aged over 50 years (64.3%) and in men (58.2%); common isolates were *E. coli* (34.8%) and MRSA (9.6%). In the multivariable logistic regression, the site of infection was associated with severe sepsis and catheter-associated UTIs^g^, significant after adjustment of age, sex, Charlson comorbidities index, and where the infection was acquired	Findings are not generalizable to other severe sepsis cohorts with bacteremia	Urinary catheter increases the risk of severe sepsis; if clinically indicated, should be used and removal dates indicated unless long-term use is required
Mo et al [16]	Cohort study	Oxfordshire, United Kingdom	4 teaching hospitals	Quantify the importance of different transmission pathways of SARS-CoV-2 in a hospital setting	For susceptible patients, 1 day in the same ward with another patient with nosocomial SARS-CoV-2 infection was associated with 7.5 infections per 1000 susceptible patients per day, while exposure to a patient with community-acquired SARS-CoV-2 infection or to an infectious health care worker had a lower risk of 2 per 1000 susceptible patients	No clarity during phase 3 onward when weekly screening was implemented. Recall bias from staff self-reporting. Staff were assumed to be absent after the first positive PCR^h^ result. Genomic sequencing of SARS-CoV-2 is not considered to confirm the transmission pathway.	Exposure to patients with nosocomial SARS-CoV-2 infection poses a risk to health care workers and hospitalized patients. Further investigation to enhance infection control and prevention around these patients is required.
Ousey et al [15]	Case- control study	England	206 patients with emergency cesarean section in an acute district general hospital	Assess the incidence risk and factors contributing to an acquired SSI^i^ after an emergency cesarean section	BMI was associated with SSI (OR 1.17, 95% CI 1.11-1.24; *P*<.001). Nonsignificant links were identified between SSIs and age and vaginal swab status.	Stigmatization of pregnant women. Use of wound swabs leading to false-positive or false-negative results. Missing or inaccurate recordings. Small sample size. Use of BMI to calculate body composition when its accuracy has been questioned.	BMI was the main risk factor for SSI because of excessive adipose tissue. Diabetes status, age, and preoperative vaginal swab status were not associated with SSI.

^a^OR: odds ratio.

^b^HAI: hospital-acquired infection.

^c^OPCS-4: Office of Population Census and Surveys.

^d^MRSA: methicillin-resistant *Staphylococcus aureus*.

^e^DSB: dry surface biofilm.

^f^MDR: multidrug resistant.

^g^UTI: urinary tract infection.

^h^PCR: polymerase chain reaction.

^i^SSI: surgical site infection.

### Themes

#### Overview

**Figure 2:**
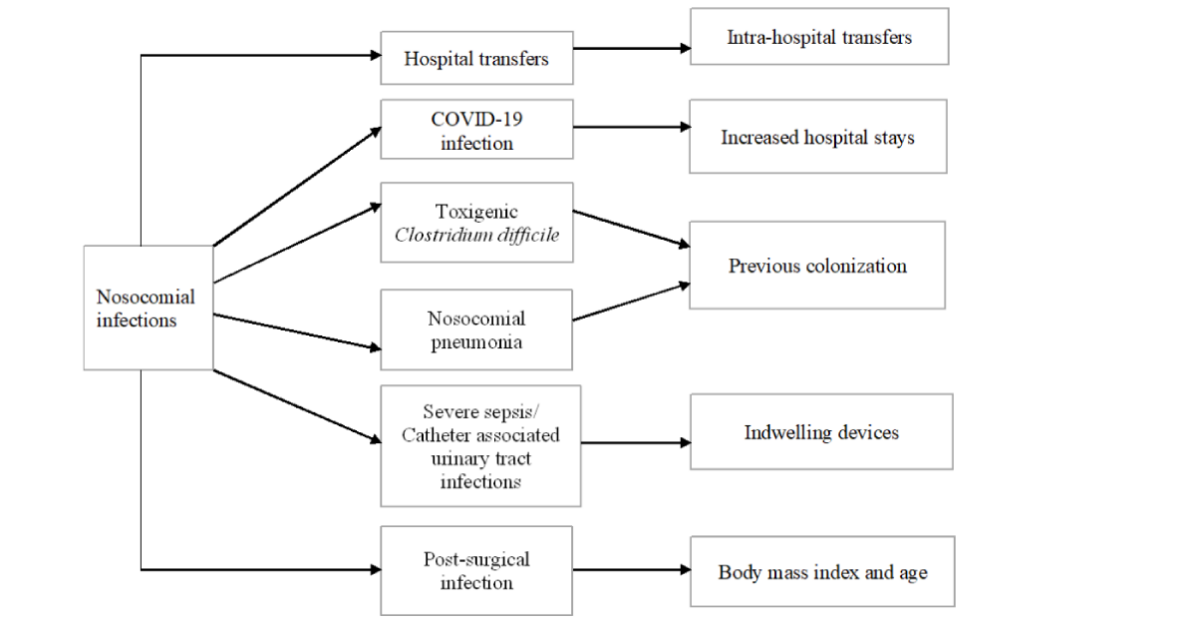
Identified themes.

The themes identified included intrahospital transfers, increased hospital stay, previous colonization, indwelling devices, and BMI and age, as shown in Figure 2.

#### Theme 1: Intrahospital Transfers

According to Boncea et al [13], older patients acquired nosocomial infections associated with ICU admissions and in-hospital deaths (*P*<.001) more frequently than younger patients 48 hours after admission (median 79, IQR 73-86 vs median 79, IQR 72-85; *P*=.004) and also had higher comorbidities (mean 4.0, SD 2.0 vs mean 3.5, SD 1.9; difference=0.5; *P*<.001). The ICU admissions cases (6.7%) and in-hospital deaths (13.3%) were significantly higher (*P*<.001) among patients 48 hours after admission. The findings demonstrated that an additional intrahospital transfer was associated with a 9% increase in the chances of developing a nosocomial infection (odds ratio [OR] 1.09, 95% CI 1.05-1.13).

Overall, 11.9% of the patients developed a nosocomial infection (49.6% men and 50.4% women) with a median age of 79 (IQR 72-86) years and a mean of 3.5 (SD 1.9) Elixhauser comorbidities, an index used to predict hospital length of stay, hospital charges, and in-hospital mortality.

In addition, 76.0% of patients with a positive lab culture collected at least 48 hours after hospitalization experienced at least one transfer compared to 71.7% of controls (patients who remained infection-free throughout the admission), with cardiology patients experiencing more movement (median 2, IQR 1-2). Logistic regression analysis was used to rule out the influence of ethnicity, gender, weekend admissions, and readmissions within 30 days. The most commonly isolated organisms from the patient group were *C. difficile*, *E. coli*, *P. aeruginosa*, *Enterococcus* species, *K. pneumoniae*, *S. aureus*, coliform species, MRSA, and coagulase-negative *Staphylococcus*.

#### Theme 2: Increased Hospital Stay

Mo et al [16] observed that an increased hospital stay increases the risk of nosocomial infections among health care workers and patients. The evidence indicated that newly admitted COVID-19 patients were associated with a risk of onward transmission to other patients in a hospital setting.

Approximately 62.1% of patients had positive SARS-CoV-2 results after admission and most of them were older. These older patients had more extended hospital stays and more readmissions. Susceptible patients were at a higher risk of acquiring COVID-19 from a patient with nosocomial COVID-19 (adjusted OR 1.76, 95% CI 1.51-2.04) than from a patient with community-acquired COVID-19 (adjusted OR 1.12, 95% CI 0.96- 1.26) or from a health care worker with COVID-19 (OR 1.45, 95% CI 1.22-1.71). These results indicated that the risk of patients transmitting COVID-19 to other patients within the same facility was higher as a result of increased hospital stay. Other infections can also be transmitted because of interacting with the same health care workers and patients for prolonged periods. Mo et al [16] observed that exposure to nosocomial infections was linked with a substantial infection risk to other hospitalized patients.

These findings are consistent with those of Ewan et al [18], who found that increased hospital stay was significantly associated with nosocomial infections (Fisher exact test *P*=.001). Similarly, Boncea et al [13] found that increased length of hospital stay was associated with nosocomial infections even when the researchers controlled for severity of illness.

#### Theme 3: Previous Colonization

According to Behar et al [17], risk factors for toxigenic *C. difficile* colonization among patients were a previous diagnosis of symptomatic *C. difficile* infection (OR 8.48, 95% CI 2.77-25.96), hospital stay of 3 months, and frailty as assessed by the Malnutrition Universal Screening Tool (MUST) and Barthel score. Multivariable analysis proved colonization to be associated with previous *C. difficile* infection (OR 4.53, 95% CI 1.33-15.48) and a MUST score indicating a higher level of frailty (≥2) (OR 3.22, 95% CI 1.47-7.06); the association with a recent hospital stay was borderline significant (OR 2.18, 95% CI 0.99-4.78).

Overall, 9.8% of the patients were carriers of *C. difficile* after their first stool test presenting with diarrhea. The most isolated ribotypes were 126 (×3), (009, 018, 020, 023, 028, 038, and 039) ×2, and ribotype 027 strains were undetected.

Ewan et al [18] found that prior carriage with opportunistic *S. aureus*, *E. coli*, *P. aeruginosa,* and MRSA (OR 9.41, 95% CI 2.28-38.78; *P*=.002) was significantly associated with nosocomial pneumonia with relative risk of 6.44 (95% CI 2.04-20.34; *P*=.002). A combination of pathogens was identified, and the highest colonization rate was identified with *Streptococcus pneumoniae* (n=27 patients) due to persistent carriage, followed by *Hemophilus influenzae* (n=8), *S. aureus* (n=6), *P. aeruginosa* (n=4), MRSA (n=3), and *E. coli* and *Acinetobacter* spp (n=2). Ewan et al [18] also identified an association between nosocomial pneumonia with admission from the hospital, active cancer, witnessed aspiration episode, increased frailty, antibiotic intake before admission, and reduced mobility.

#### Theme 4: Indwelling Devices

Melzer and Welch [19] sought to establish whether the presence of a urinary catheter predicts severe sepsis as a nosocomial infection within a bacteremic cohort. The researchers identified 64.3% bacteremic episodes in patients >50 years old, 58.2% of whom were men. The episodes were community-acquired, health care–associated, and hospital-acquired infections, but community-acquired episodes were not associated with severe sepsis (Pitt score≥2). Site of infection, individual patient, critical care admission, and catheter-associated UTI were significantly associated with severe sepsis. After multivariate analysis, the site of infection was significantly associated with severe sepsis, with a stronger association for catheter-associated UTIs (OR 3.87, 95% CI 1.82-8.22), which remained significant after adjustment for age and where the infection was acquired (OR 3.94, 95% CI 1.70-9.11). These results indicate that indwelling devices such as a urinary catheter are a risk factor that can predict nosocomial infections such as severe sepsis.

#### Theme 5: BMI and Age

Wloch et al [20] found that 10 days following a hospital admission was the median time for all SSIs and 8 days of admission was the median time for deep and organ infections alone. The mentioned risk factors for these nosocomial infections included BMI, which was strongly associated with superficial infections (*P*<.001), and deep organ space (*P*<.003). After multivariate analysis, women with a BMI of 25-30 (overweight) had an estimated OR of 1.6 (95% CI 1.2-2.2) compared to that of women within a normal BMI range. The results also indicated that women with a BMI >30 (obese) had a 2.4-times greater chance of nosocomial infection (95% CI 1.7-3.4).

After multivariate analysis, age had a stronger association for SSIs and women <20 years of age (*P*=.04) had a weaker association for SSIs. *S. aureus* was the commonly associated organism at 40.4%; 17.7% were methicillin-resistant organisms and the rest included anaerobic cocci at 23.2%, Enterobacteriaceae at 13.3%, and streptococci at 7.4%.

Boncea et al [13] observed that increased age was a risk factor for nosocomial infections in a study focused on hospital-acquired infections in an urban UK hospital network. Patients with hospital-acquired infections were older than those who did not acquire the infections (median 79, IQR 73-86 vs median 79, IQR 72-85; *P*<.001). Boncea et al [13] also found that older patients had a higher number of comorbidities than younger patients (mean 4.0, SD 2.0 vs mean 3.5, SD 1.9; difference=0.5; *P*<.001).

## Discussion

### Principal Findings

The findings of this systematic review provided answers to the study objectives. The main risk factors of nosocomial infections identified in this study include intrahospital transfers, increased hospital stay, previous colonization, indwelling devices, and BMI and age.

Blay et al [22] demonstrated that intrahospital transfers increased the risk of developing nosocomial infections, with the majority of patients in the study undergoing 2.4 transfers per year. This finding has been supported by Boncea et al [13], who reported that an additional intrahospital transfer was associated with a 9% higher risk of acquiring infections in a hospital. Boncea et al [13] also found that older patients were more likely to develop nosocomial infections. The prevalence of nosocomial infections was high in the older population due to frailty and immunosuppression. Intrahospital transfers occurred as a result of lesser equipped wards along with procedure rooms and bed shortages that expose susceptible patients to infectious hospital surfaces, staff, and other patients, thus increasing their risk of nosocomial infection. The findings suggested that minimizing infection risk to be a priority when transferring patients. Kulshrestha and Singh [23] mentioned that patient intratransfers should aim at maintaining patient care to minimize morbidity and mortality. Single-patient rooms can be used to lower pathogen transmission and the number of intrahospital transfers, thereby reducing interactions between staff, hospital surfaces, and patients to eventually reduce the incidence of nosocomial infections [13].

Mo et al [16] observed that COVID-19 transmission between patients and staff had a stronger association for nosocomial COVID-19 than community-acquired COVID-19 [16]. The daily risk of COVID-19 was associated with increased exposure to newly infected patients and staff. Medical professionals can address this challenge by providing adequate sick pay leave for symptomatic staff for better complete recovery and regular screening for early detection to reduce cross-infection and control for the incidence of nosocomial COVID-19. Furthermore, early symptom presentation and regular screening of staff and patients is a better strategy for infection prevention and control, where infected patients and staff should be isolated immediately.

According to Behar et al [17], older patients were largely colonized by *C. difficile* presenting with diarrhea and contributing to transmission to other patients. The risk of colonization with asymptomatic *C. difficile* was due to frailty associated with suppressed immunity, which resulted in increased mortality. The risk of colonization was also associated with previous diagnoses and longer hospital stays. Early detection and isolation upon admission can reduce the further spread of nosocomial *C. difficile*. In a study by Dubberke et al [24], patients showed acquisition of *C. difficile* while hospitalized. The initial intake of antibiotics was associated with disease, whereas susceptibility to colonization with *C. difficile* was associated with suppressed immunity and an altered intestinal flora composition, gender, preexisting comorbidities, and frailty-determined mortality rate [17]. Risks such as previous hospital admission, antibiotic therapy, and immune suppression were well documented and explained in a similar study on *C. difficile* infection in hospitalized children in the United States [25].

Nosocomial pneumonia occurred after admission, with most samples positive for colonization with *S. aureus*, MRSA, *P. aeruginosa,* and *E. coli*, which resulted in extended hospital stays of 30 days, similar to patients with ventilator-associated pneumonia and aspiration pneumonia in medical patients [18]. Colonization was identified as the key risk factor for nosocomial pneumonia.

The results of this systematic review indicate that detection using molecular techniques resulted in a high incidence rate of nosocomial pneumonia in the exposed patient group. Ewan et al [13] failed to identify *K. pneumoniae* and Enterobacteriaceae as prevalent organisms and a risk of nosocomial pneumonia in frailer and unwell patients [26]. Oral colonization began within 72 hours of admission in a majority of the cases, with the possible explanation that some have been acquired from the community or upon admission; therefore, interventions should start within this timeframe. Delivery of better oral hygiene at the ward level in a resource-scarce health system can control for antibiotic use and length of hospital stay associated with nosocomial pneumonia.

Melzer and Welch [19] identified the presence of urinary catheters to be strongly associated with severe sepsis, with a stronger association for gram-negative multidrug-resistant *E. coli*. Furthermore, underlying diseases and obstruction history are also identifiable risks for severe sepsis. *E. coli*, a gram-negative bacterium gastrointestinal colonizer, is known to commonly cause UTIs and septicemia [26], which was also associated with delayed administration of antibiotics. To account for this, patients exhibiting any signs of sepsis should be given immediate admission or management in critical care areas, and implementation of a critical care bundle approach would result in reduced admissions due to severe sepsis. A critical care bundle would aid in the documentation of reasons for catheter insertion, removal dates, and aseptic techniques during and after insertion to control for infection. Medical professionals can increase patient satisfaction and lower infection risks by issuing catheter passports to long-term users, guiding community nurses on the use of prophylactic antibiotics when changing catheters, and using suprapubic catheters.

SSIs were strongly associated with an inpatient stay, readmissions, diabetes, surgeons’ grade, and women <20 years and >45 years of age. The most commonly isolated organisms from the skin or genital flora were *S. aureus* (40.4%, 17.1% of which were MRSA), anaerobic cocci (23.2%), Enterobacteriaceae (13.3%), and streptococci (7.4%). BMI was strongly associated with SSIs due to poor prescription techniques that exclude patients’ BMI, longer incision time, and slow transit of antibiotics due to thick adipose tissue that result in impaired wound healing and decreased immunity. This view on BMI has been supported by Ousley et al [15]. According to Chu et al [27], younger age in women is associated with an increased risk for SSIs due to underdeveloped immunity to fight infections. Older age is associated with frailty and a suppressed immune system, which has been supported by Boncea et al [13], who also mentioned readmission and increased hospital stay resulting in exposure of patients to hospital surfaces with pathogenic organisms, thereby increasing the risk for nosocomial infections. Another study confirmed diabetes to be a risk of postsurgical infections, which is a marker for other conditions such as vascular changes and white blood cell malfunction, resulting in suppressed immunity that exposes a patient to multiple infections during and after surgery [28]. In general, the analyzed studies identified impacts of nosocomial infections to be associated with increased admissions, increased hospital stay, and costs on health care services. Other studies have reached similar findings and identified that older age, readmissions, and longer hospital stay are associated with increased infection risk due to exposure to infectious pathogens, frailty, and suppressed immunity. Özdemir and Dizbay [29] found that older patients have a higher risk of nosocomial infections resulting from impaired immune defense, long-term hospital stay, immunosuppressive treatments, and underlying chronic illnesses.

### Study Strengths and Weaknesses

There were no systematic reviews on risk factors of nosocomial infections in hospitalized patients, and the study identified both qualitative and quantitative data relevant to the research topic. As another strength, this review pointed out key risk factors associated with nosocomial infections and the patient populations at risk, additionally suggesting measures for infection control and prevention, providing an advantage to the NHS whose priority is controlling health care–associated infections [30]. In this review, non-English articles were excluded, which may have limited capturing information in other languages.

### Conclusions

Patients with previous colonization and underlying conditions are at increased risk of nosocomial infections due to frailty, extended hospital stay, and suppressed immunity. The use of indwelling devices, increased age, high BMI, and intrahospital transfers increase the susceptibility of patients to nosocomial infections.

Intrahospital transfers expose patients to other infectious patients, staff, and hospital environments, thereby increasing their risk for infection. In addition, the number of staff per patient has been positively associated with an increased risk of infection. Readmissions, underlying conditions and/or active cancer, prior antibiotic intake associated with drug-resistant microorganisms, and colonization with opportunistic organisms contribute to nosocomial infections. Indwelling devices such as urinary catheters increase the risk of sepsis, while obesity and age are strongly associated with SSIs. The most common organisms associated with nosocomial infections included *C. difficile*, *E. coli*, *P. aeruginosa*, *Enterococcus* spp, *K. pneumoniae*, *S. aureus*, coliform species, anaerobic cocci, MRSA, coagulase-negative *Staphylococcus*, Enterobacteriaceae, and streptococci.

Implemented interventions should consider the time frame before and after admission to control for organisms acquired during both periods. Further studies are needed to identify the risk factors of nosocomial infections and drug-resistant pathogens targeting a larger patient group for better infection prevention and control.

## Appendix

Multimedia Appendix 1 PRISMA 2020 Checklist.

## Abbreviations

AXIS: Appraisal Tool for Cross-Sectional Studies
CASP: Critical Appraisal Skills Programme
ICU: intensive care unit
MRSA: methicillin-resistant Staphylococcus aureus
MUST: Malnutrition Universal Screening Tool
NHS: National Health Service
OR: odds ratio
PRISMA: Preferred Reporting Items for Systematic Reviews and Meta-Analyses
SSI: surgical site infection
UTI: urinary tract infection

## References

[1] Samuel S, Kayode O, Musa O, Nwigwe G, Aboderin A, Salami T, Taiwo S (2010). Nosocomial infections and the challenges of control in developing countries. Af J Clin Exp Micro.

[2] Lobdell KW, Stamou S, Sanchez JA (2012). Hospital-acquired infections. Surg Clin North Am.

[3] Kaye KS, Marchaim D, Chen T, Baures T, Anderson DJ, Choi Y, Sloane R, Schmader KE (2014). Effect of nosocomial bloodstream infections on mortality, length of stay, and hospital costs in older adults. J Am Geriatr Soc.

[4] Llewellyn C, Ayers S, McManus C, Newman S, Petrie K, Revenson T (2019). Cambridge Handbook of Psychology, Health and Medicine, 3rd edition.

[5] Sadique Z, Lopman B, Cooper BS, Edmunds WJ (2016). Cost-effectiveness of ward closure to control outbreaks of norovirus infection in United Kingdom National Health Service hospitals. J Infect Dis.

[6] Freeman J, McGowan JE (1978). Risk factors for nosocomial infection. J Infect Dis.

[7] Forster AJ, Taljaard M, Oake N, Wilson K, Roth V, van Walraven C (2012). The effect of hospital-acquired infection with Clostridium difficile on length of stay in hospital. CMAJ.

[8] Sheldon L, Krieger R (2010). Chapter 42: Exposure framework. Hayes' handbook of pesticide toxicology, 3rd edition.

[9] Page MJ, McKenzie JE, Bossuyt PM, Boutron I, Hoffmann TC, Mulrow CD, Shamseer L, Tetzlaff JM, Akl EA, Brennan SE, Chou R, Glanville J, Grimshaw JM, Hróbjartsson A, Lalu MM, Li T, Loder EW, Mayo-Wilson E, McDonald S, McGuinness LA, Stewart LA, Thomas J, Tricco AC, Welch VA, Whiting P, Moher D (2021). The PRISMA 2020 statement: an updated guideline for reporting systematic reviews. BMJ.

[10] Braun V, Clarke V (2006). Using thematic analysis in psychology. Qual Res Psychol.

[11] Long HA, French DP, Brooks JM (2020). Optimising the value of the critical appraisal skills programme (CASP) tool for quality appraisal in qualitative evidence synthesis. Res Meth Med Health Sci.

[12] Ma L, Wang Y, Yang Z, Huang D, Weng H, Zeng X (2020). Methodological quality (risk of bias) assessment tools for primary and secondary medical studies: what are they and which is better?. Mil Med Res.

[13] Boncea EE, Expert P, Honeyford K, Kinderlerer A, Mitchell C, Cooke GS, Mercuri L, Costelloe CE (2021). Association between intrahospital transfer and hospital-acquired infection in the elderly: a retrospective case-control study in a UK hospital network. BMJ Qual Saf.

[14] Alrawi M, Crowley TP, Pape SA (2014). Bacterial colonisation of the burn wound: a UK experience. J Wound Care.

[15] Ousey K, Blackburn J, Stephenson J, Southern T (2021). Incidence and risk factors for surgical site infection following emergency cesarean section: a retrospective case-control study. Adv Skin Wound Care.

[16] Mo Y, Eyre DW, Lumley SF, Walker TM, Shaw RH, O'Donnell D, Butcher L, Jeffery K, Donnelly CA, Cooper BS, Oxford COVID Infection Review Team (2021). Transmission of community- and hospital-acquired SARS-CoV-2 in hospital settings in the UK: a cohort study. PLoS Med.

[17] Behar L, Chadwick D, Dunne A, Jones CI, Proctor C, Rajkumar C, Sharratt P, Stanley P, Whiley A, Wilks M, Llewelyn MJ (2017). Toxigenic Clostridium difficile colonization among hospitalised adults; risk factors and impact on survival. J Infect.

[18] Ewan VC, Sails AD, Walls AWG, Rushton S, Newton JL (2015). Dental and microbiological risk factors for hospital-acquired pneumonia in non-ventilated older patients. PLoS One.

[19] Melzer M, Welch C (2017). Does the presence of a urinary catheter predict severe sepsis in a bacteraemic cohort?. J Hosp Infect.

[20] Wloch C, Wilson J, Lamagni T, Harrington P, Charlett A, Sheridan E (2012). Risk factors for surgical site infection following caesarean section in England: results from a multicentre cohort study. BJOG.

[21] Ledwoch K, Dancer SJ, Otter JA, Kerr K, Roposte D, Maillard J (2021). How dirty is your QWERTY? The risk of healthcare pathogen transmission from computer keyboards. J Hosp Infect.

[22] Blay N, Roche MA, Duffield C, Gallagher R (2017). Intrahospital transfers and the impact on nursing workload. J Clin Nurs.

[23] Kulshrestha A, Singh J (2016). Inter-hospital and intra-hospital patient transfer: recent concepts. Indian J Anaesth.

[24] Dubberke ER, Reske KA, Seiler S, Hink T, Kwon JH, Burnham CD (2015). Risk factors for acquisition and loss of Clostridium difficile colonization in hospitalized patients. Antimicrob Agents Chemother.

[25] Nylund CM, Goudie A, Garza JM, Fairbrother G, Cohen MB (2011). Clostridium difficile infection in hospitalized children in the United States. Arch Pediatr Adolesc Med.

[26] Khan HA, Ahmad A, Mehboob R (2015). Nosocomial infections and their control strategies. Asian Pacific J Trop Biomed.

[27] Chu K, Maine R, Trelles M (2015). Cesarean section surgical site infections in sub-Saharan Africa: a multi-country study from Medecins Sans Frontieres. World J Surg.

[28] Martin ET, Kaye KS, Knott C, Nguyen H, Santarossa M, Evans R, Bertran E, Jaber L (2016). Diabetes and risk of surgical site infection: a systematic review and meta-analysis. Infect Control Hosp Epidemiol.

[29] Özdemir K, Dizbay M (2015). Nosocomial infection and risk factors in elderly patients in intensive care units. J Microbiol Infect Dis.

[30] Duerden B (2008). Controlling healthcare-associated infections in the NHS. Clin Med.

